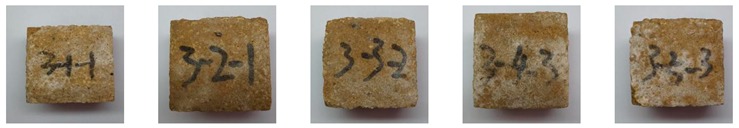# The Effect of Nano-Particles and Water Glass on the Water Stability of Magnesium Phosphate Cement Based Mortar

**DOI:** 10.3390/ma12223755

**Published:** 2019-11-14

**Authors:** Hu Feng, Xiangyu Zhao, Gang Chen, Changwei Miao, Xiaocong Zhao, Danying Gao, Gangzhu Sun

**Affiliations:** 1 School of Civil Engineering, Zhengzhou University, Zhengzhou 450001, China; fenghu@zzu.edu.cn (H.F.); zhaoxiangyu1208@126.com (X.Z.);; 2 College of Civil Engineering, Henan University of Engineering, Zhengzhou 451191, China; 3 China Nuclear Industry Geotechnical Engineering Co., Ltd., Zhengzhou 451191, China

**Keywords:** magnesium phosphate cement, nano-particles, water glass, water stability

## Abstract

This paper experimentally presented the water stability of magnesium phosphate cement (MPC) modified by nano-Al_2_O_3_ (NA), nano-Fe_2_O_3_ (NF) and water glass (WG). The optimal addition of 6% NA, 2% NF and 1% WG significantly improved the water stability of MPC mortar by 86%, 101% and 96% after 28 days of water immersion, respectively. X-Ray Diffraction (XRD) and Scanning Electron Microscope (SEM) were used to analyze the water stability of MPC modified by NA, NF and WG. The results of the micrograph and composition analysis revealed that the proper amount of NA, NF or WG could fill the micro pores and improve the hydration of interior structures of MPC mortar. Thus, the microstructural compactness was satisfied to keep a good water stability of MPC mortar.

## 1. Introduction

The Magnesium Phosphate Cement (MPC) is a fast-hardening gelling material produced by a hydration reaction of re-burned magnesia (MgO) and phosphate (KH_2_PO_4_) [1]. Compared with ordinary Portland cement, it has outstanding advantages: (1) high early strength and fast setting and hardening, (2) strong adhesion and (3) good corrosion resistance and freezing resistance [2,3,4]. Therefore, it is widely used in the repair of roads, bridges and airstrips, waste solidification and fiber reinforced composite material external reinforcement technology [5,6,7]. Studies have shown that MPC mortar will shrink when immersed in water [8,9,10,11]. This phenomenon has a negative impact on the wide application of MPC mortar [12]. Therefore, improving water resistance is necessary for promoting the application of MPC mortar. The evaluation indexes of water resistance of MPC include: (1) the softening coefficient, (2) mass loss rate, (3) strength retention rate, (4) potassium magnesium phosphate retention rate and (5) attenuation rate [3,13,14,15,16]. The strength retention rate is intuitive and easy to operate [11]. Regarding the research on the water resistance of MPC, it has been shown that the addition of water glass effectively improves the water resistance of the MPC-based material [17,18,19,20,21,22]. The incorporation of water glass accelerates the early hydration reaction rate of MPC and increases its early compressive strength [23,24]. Secondly, water glass can react with MgO to form a hydrated magnesium silicate gel [25,26]. The gel can be filled in the pores of the hardened body, hindering the immersion of the external aqueous solution [27,28], and effectively reducing the dissolution of unreacted KH_2_PO_4_ and hydration products, thereby improving the water resistance of the material. According to the relevant literature, the compressive strength of the MPC mortar test for natural curing for 1 day and 7 days is about 80% and 90% of the compressive strength of 28 days respectively [29,30,31]. The hydration reaction can be completed in one day. Since the 7 days compressive strength and the 28 days compressive strength are very close, it is reasonable to study the water stability of the MPC mortar after room temperature curing for 7 days, and then immersing in water.

The reasons for the poor water resistance of MPC mortar are as follows: (1) The main components in the test piece are MgO and KH_2_PO_4_, which are not involved in the reaction, and the main product of the reaction (potassium magnesium phosphate hexahydrate, MgKPO_4_·6H_2_O). After immersing in water, the KH_2_PO_4_, which was not completely reacted, was further dissolved and precipitated along the pores and fine cracks inside the test piece, and recrystallized on the surface of the test piece [11,32,33]. Therefore, the porosity inside the test piece is further increased. (2) The dissolved KH_2_PO_4_ and the surplus MgO are further reacted to generate a great tension at the micro cracks, which also causes a decrease in compressive strength [34]. The pH value of the solution has a great influence on the hydration reaction of MPC [35,36,37]. The test piece is placed in water due to the large amount of unreacted KH_2_PO_4_ dissolved to cause a weak acid environment, which promotes the decomposition of the hydration reaction product [38,39,40]. The solution tends to forming a weak alkaline environment and reaching a relatively stable pH value with the gradual dissolution of the product [41,42,43]. This dynamic process leads to a decrease in compactness and compressive strength.

In recent years, the application of nano-particles in the concrete field has been gradually expanded [23,44,45]. It has the characteristics of small particles, large specific surface area and high activity. The nanometer scale of nano-particles plays a positive role in improving the performance of traditional concrete. It was found that nano-particles could improve pore structure, reduce porosity and increase structural compactness for ordinary Portland cement [45,46,47,48]. Therefore, the nano-particles have the potential to improve the properties of MPC based composites. However, little research has been done on the effect of nano-particles on the mechanical properties and water stability of MPC.

This paper describes the strength and water stability of MPC mortar with the addition of nano-particles. The variables investigated include mixture design parameter, the types of nano-particles and the amount of nano-particles, curing time and curing condition. Also, the compressive strength and the strength retention rate characteristics of MPC mortar mixed with nano-Al_2_O_3_, nano-Fe_2_O_3_ and water glass were analyzed.

## 2. Experimental Program

### 2.1. Materials

The magnesium phosphate cement (MPC) was prepared from a mixture of re-burned magnesia (MgO, labelled as M), potassium dihydrogen phosphate (KH_2_PO_4_, labelled as P) and retarder (Na_2_B_4_O_7_∙10H_2_O, labelled as B). The summary of M, P and B is described in Table 1. Nano- Al_2_O_3_ (labelled as NA), nano-Fe_2_O_3_ (labelled as NF) and water glass (Na_2_SiO_3_∙5H_2_O, labelled as WG) were used as admixtures and are summarized in Table 1. Standard sand conformed to ISO 679-2009 [49] was used as fine aggregates. Tap water was used to mix the mixtures.

### 2.2. Mixture Proportions

The MPC mortar was used for water stability evaluation and Scanning Electron Microscope (SEM) observation. The MPC paste without fine aggregates was used for X-Ray Diffraction (XRD) analysis. It is considered that the incorporation of fine aggregates would emphasize the high spectrum peaks of SiO_2_ while weakening the concerned spectrum peaks of other compositions. The standard sand in the MPC mortar does not participate in hydration reaction. Therefore, the cement paste is favorable and recommended for the composition analysis. [55,56] The variables are weight ratio of water to binder (*w*/*b*), weight ratio of fine aggregate to binder (*s*/*b*), mole ratio of M to P (*m*/*p*). The binder is the combination of MPC, NA, NF and/or WG. The MPC mortar and MPC paste follow the same mixture proportions, except the MPC paste is without fine aggregates. The mixture proportions of MPC mortar and paste are listed in Table 2. The dosages of NA, NF and WG in Table 2 are represented by the weight replacement ratio of MPC.

### 2.3. Specimens Preparation

Firstly, the M, P, B and fine aggregates (without fine aggregates for MPC paste) were mixed by a 20 L concrete mixer (HX-15, Huida, Tianjin, China) with single horizontal shaft at a low speed of 60 s. Secondly, the NA, NF and/or WG were slowly added into the mixture. Then, the water was added into the mixer and mixed for another 60 s, followed by a rapid mixing for 60 s. All mixed mixtures were moulded into big cubes of 50 mm × 50 mm × 50 mm for water stability evaluation, small cubes of 40 mm × 40 mm × 40 mm for XRD analysis and columns of 25 mm (diameter) × 30 mm (height) for SEM observation. All specimens were demolded after 2 h and cured in a curing room (temperature 15–20 °C, relative humidity 40%–50%) for 7 days to achieve a full hydration and stable hydration strength [57]. The curing room provided a stable environment of temperature and humidity to guarantee the fast hydration of the MPC. After 7 days of curing, the hydration reaction was basically completed, and the increase of compressive strength was minor and stable. Afterwards, the big cubes were immersed in water (temperature 20–25 °C) for another 7 days or 28 days, while the small cubes and columns of typical groups were only immersed for another 7 days, as illustrated in Table 2. For each group, nine big cubes (each three for 0 days, 7 days and 28 days of water immersion) were prepared for water stability evaluation; one small cube, or both of one small cube and one column of each typical group were prepared for XRD analysis and SEM observation, respectively. Thus, a total of 243 big cubes, 12 small cubes and 9 columns for 47 groups, were prepared. The grouped specimens were described in Table 2. The appearance of prepared big cubes with NA, NF and WG are shown in Table 3, Table 4 and Table 5, respectively.

Before XRD analysis and SEM observation, the prepared small cubes and columns were immersed in absolute ethanol, and dried in a vacuum oven with 45 °C for 48 h to inhibit subsequent hydration. The XRD analysis requires the fine powder sample. Therefore, the prepared small cubes were crushed into a powder and sieved by a standard sieve of 325 meshes. Then, the powder was bottled and labelled. In order to match the space of the SEM sample stage, the prepared columns were cut into slices with a thickness of 1.5–2 mm, and then broken into small pieces.

### 2.4. Test Procedure

The water stability is evaluated by the retention ratio of the compressive strength of the specimens before and after water immersion, thus, the test of compressive strength is needed. The test was conducted by a universal testing machine (WDW-100, Docer, Jinan, China) with a capacity of 2000 kN and follows the standard of ASTM C109 [58]. 

The XRD analysis was achieved by the XRD of Panalytical X’Pert3 Powder (Malvern Panalytical, Almelo, the Netherlands), at a scanning range 10°–90°, step size 0.03 and speed of 10°–20°/min. The SEM observation was achieved by the SEM of Zeiss Auriga (Zeiss, Oberkochen, Germany).

## 3. Results and Discussion

### 3.1. Water Stability Evaluation

The water stability Kt is described by the retention ratio of the compressive strength of the specimens before and after water immersion and is written, as following [59],

(1) $$Kt=\frac{f}{F}\times 100\%,$$

where f and F are the compressive strength of specimens with and without water immersion, respectively. All displayed test results were represented by the average value of three specimens for each group and immersion time.

#### 3.1.1. Effect of *w*/*b*, *s*/*b* and *m*/*p* on the Water Stability of MPC Mortar

**Figure 1:**
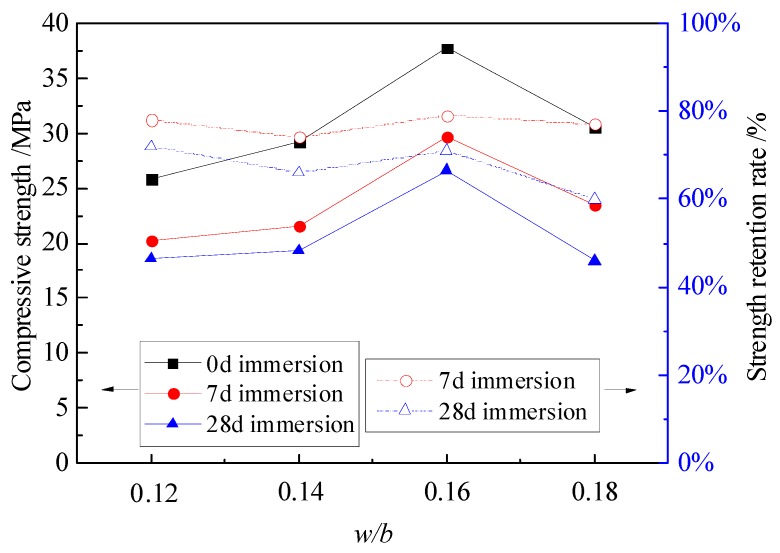
Compressive strength and strength retention rate of specimens with different *w/b*.

Figure 1 shows the compressive strength and strength retention rate curves of MPC mortar with different *w*/*b* ratios. With the increase of *w*/*b* from 0.12 to 0.18, the compressive strength showed a curve with one peak. The maximum compressive strength occurred at *w*/*b* = 0.16, that is 37.7 MPa, 29.7 MPa and 26.6 MPa for specimens without immersion, with 7 days of immersion and with 28 days of immersion, respectively. The Kt moderately reduced to 74.0% and 66.1% for specimens with 7 days and 28 days of immersion at *w*/*b* = 0.14, respectively. However, increasing the *w*/*b* from 0.14 to 0.16, the Kt of specimens with 7 days and 28 days of immersion gradually increased to 78.8% and 70.6% at *w*/*b* = 0.16, and then decreased to 76.7% and 60.3% at *w*/*b* = 0.18. Since a balance of *w/b* exists in the work ability of fresh mortar and compressive strength of hardened mortar, the 0.16 is a reasonable *w*/*b* to guarantee desirable work ability as well as a good compactness of the interior structure of MPC mortar. Therefore, the maximum compressive strength and Kt were achieved at *w*/*b* = 0.16.

**Figure 2:**
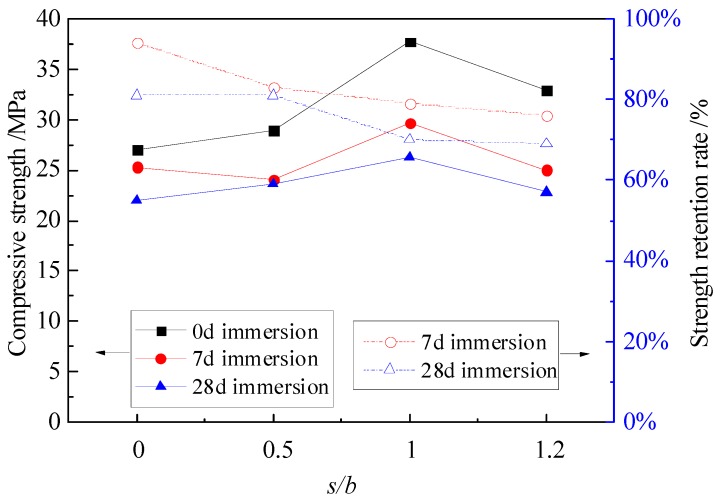
Compressive strength and strength retention rate of specimens with different *s/b*.

Figure 2 shows the compressive strength and strength retention rate curves of MPC mortar with different *s*/*b* ratios. The *s*/*b* ratio plays a dominant role in the fluidity of mortar. Fluidity is closely related to the interior structure of hardened MPC mortar. The best fluidity was observed in the specimens with *s*/*b* = 1.0 in this condition, the compressive strength was reached to the maximum of 37.7 MPa, 29.7 MPa and 26.3 MPa for specimens without immersion, with 7 days of immersion and with 28 days of immersion, respectively. However, increasing *s*/*b* from 0 to 1.2 resulted in a descent trend of strength retention rate. Compared to specimens with *s*/*b* = 0, the strength retention rates of specimens with *s*/*b* = 1.2 were decreased by 18% and 12% after 7 days and 28 days of immersion, respectively. Therefore, it can be deduced that the higher *s*/*b* exerted more negative effect on the interior structure as well as the water stability of the MPC mortar. 

**Figure 3:**
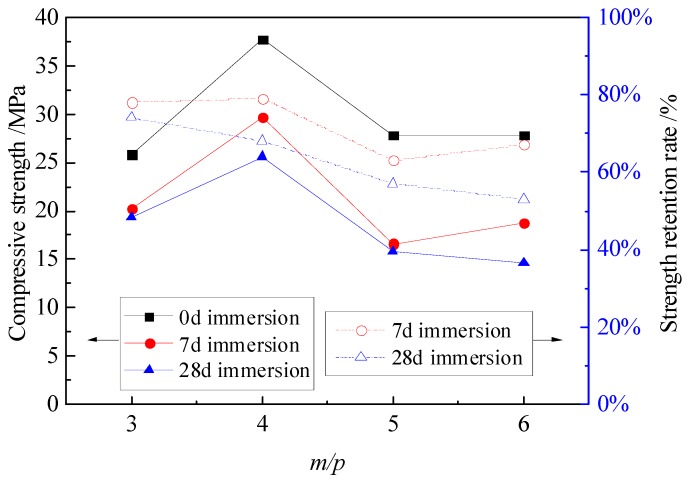
Compressive strength and strength retention rate of specimens with different *m/p*.

Figure 3 shows the compressive strength and strength retention rate curves of the MPC mortar specimens with different *m*/*p*. Increasing the *m*/*p* up to 4 led to an expansion effect on the interior structures of the MPC mortar, and thus the compressive strength was improved to 37.7 MPa, 29.7 MPa and 25.6 MPa for specimens without immersion, with 7 days of immersion and with 28 days of immersion. When *m*/*p* increased from 4 to 6, the expansion effect was diminished, while the drying shrinkage effect occurred. The drying shrinkage effect was caused by the accelerated setting time at curing, which eventually resulted in a poor interior structure of hydration product of the MPC mortar. For this reason, the compressive strength of the specimens with or without immersion was dramatically reduced, even close to or lower than that of specimens with *m*/*p* = 3. The *m*/*p* has a strong effect on drying shrinkage and setting time during the curing of the MPC mortar. Thus, the increase of *m*/*p* will accelerate the setting time and results in a poor interior structure of hydration product of the MPC mortar. Although the expansion effect is more influential on compressive strength, the strength retention rate was not significantly influenced at *m*/*p* for specimens with 7 days and 28 days of immersion, and generally showed a downward trend with *m*/*p* up to 6.

#### 3.1.2. Effect of NA, NF and WG Dosage on the Water Stability of MPC Mortar

**Figure 4:**
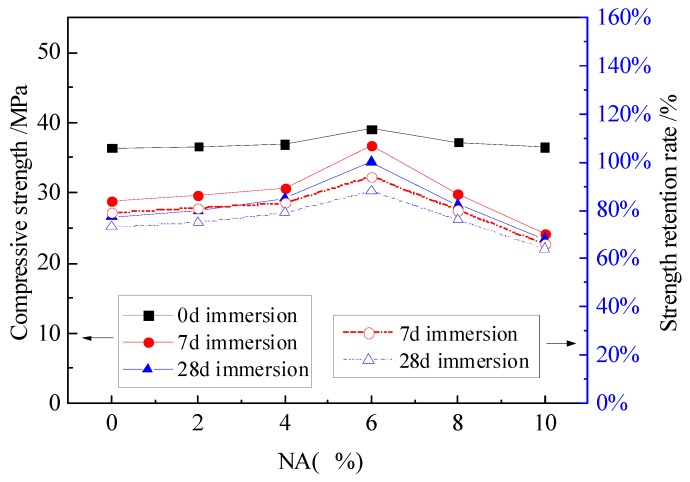
The effect of NA on the compressive strength and strength retention rate.

**Figure 5:**
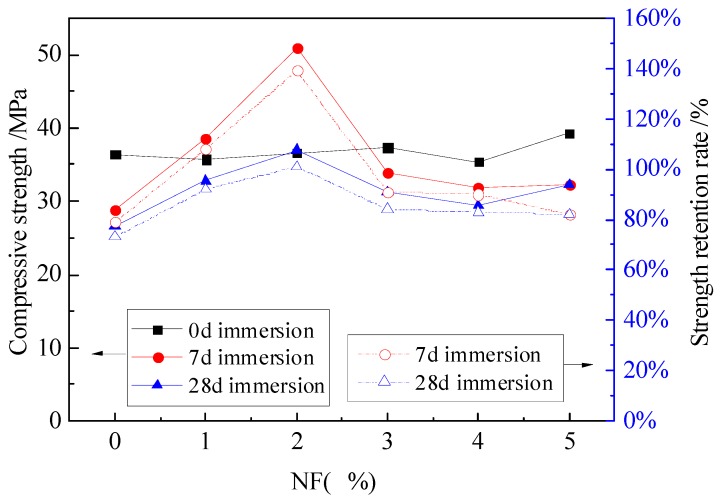
The effect of NF on the compressive strength and strength retention rate.

**Figure 6:**
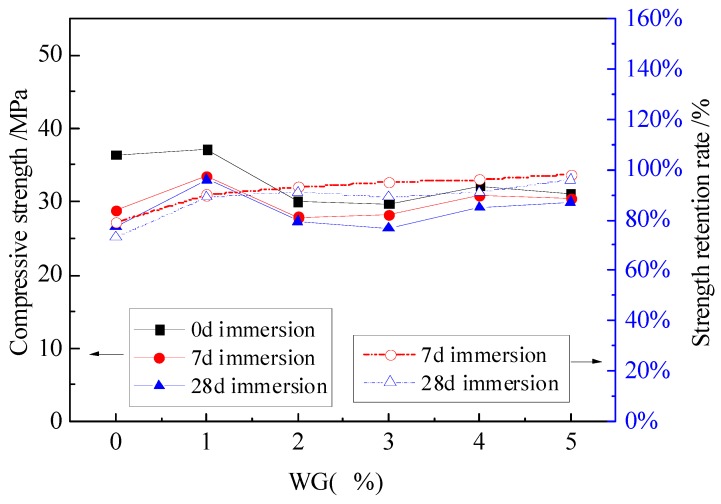
The effect of WG on the compressive strength and strength retention rate.

The compressive strength and strength retention rate curves of MPC mortar with different dosages of NA and NF are shown in Figure 4, Figure 5 and Figure 6.

Figure 4 shows that a small dosage of NA, within 4%, has little effect on the compressive strength and strength retention rate. However, when the NA dosage increased from 4% to 6%, the compressive strength was significantly improved to 39.1 MPa, 36.7 MPa and 34.4 MPa for specimens without immersion, with 7 days of immersion and with 28 days of immersion, respectively; while the strength retention rates were improved to 94% and 88% for specimens with 7 days and 28 days of immersion.

As Figure 5 indicates, the addition of NF has little effect on the compressive strength of specimens without immersion, but 2% NF would greatly improve the compressive strength and strength retention ratio for specimens with 7 days and 28 days of immersion. Compared to specimens without NF, a total of 60% and 28% increase in compressive strength were noticed for specimens with 7 days and 28 days of immersion, respectively. A similar increase was also found in the strength retention ratio. However, with 3% or more NF used, the specimen performed a sharp decrease in compressive strength and strength retention, which are little higher than specimens without NF.

The obvious increase of compressive strength only exists in the specimens with 1% WG. The increased compressive strength is 33.4 MPa and 33 MPa for specimens with 7 days and 28 days of immersion, respectively, as shown in Figure 6, but the increase was reduced when 2% WG was added. Increasing WG from 2% to 5% appeared to have little effect on the compressive strength, which is similar with the results of specimens without WG. Nevertheless, the strength retention ratio always increased for specimens with WG from 0% to 5%, but the increase is implicit for specimens with 3% to 5% WG. Compared to specimens without WG, a total of 19% increase was obtained for specimens with WG up to 5%. Therefore, with the consideration of economy and performance, 1%–3% WG tends to be suitable to improve the compressive strength and water stability of the MPC mortar.

Above all, appropriate dosage of NA, NF and WG has improvement in compressive strength and water stability to some extent, compared with the specimens without any admixtures. For specimens with NF, the compressive strength is a litter higher than that of specimens with NA and WG. Moreover, the specimens with NF have a higher strength retention rate than those with NA and WG. Therefore, NF plays an important role to improve the water stability.

### 3.2. Compositions Analysis Based on XRD Tests

**Figure 7:**
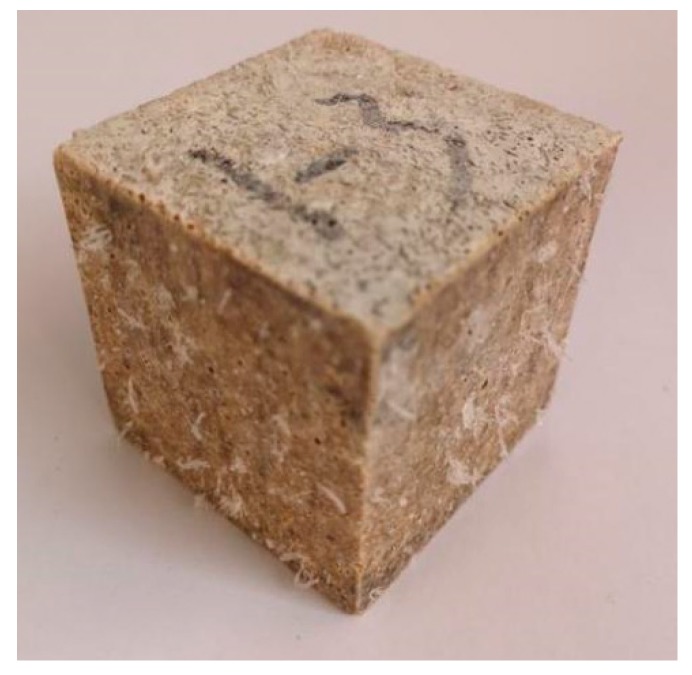
MPC mortar specimen of 7 days immersion.

On the third day of immersion, deposits of white needle-like crystals began to appear on the surface of small cubes and were suspended in the water. Then, the amount of deposits increased until the seventh day, as shown in Figure 7. The deposits on the cube surface and the powder crushed from the small cubes were collected for the XRD analysis.

**Figure 8:**
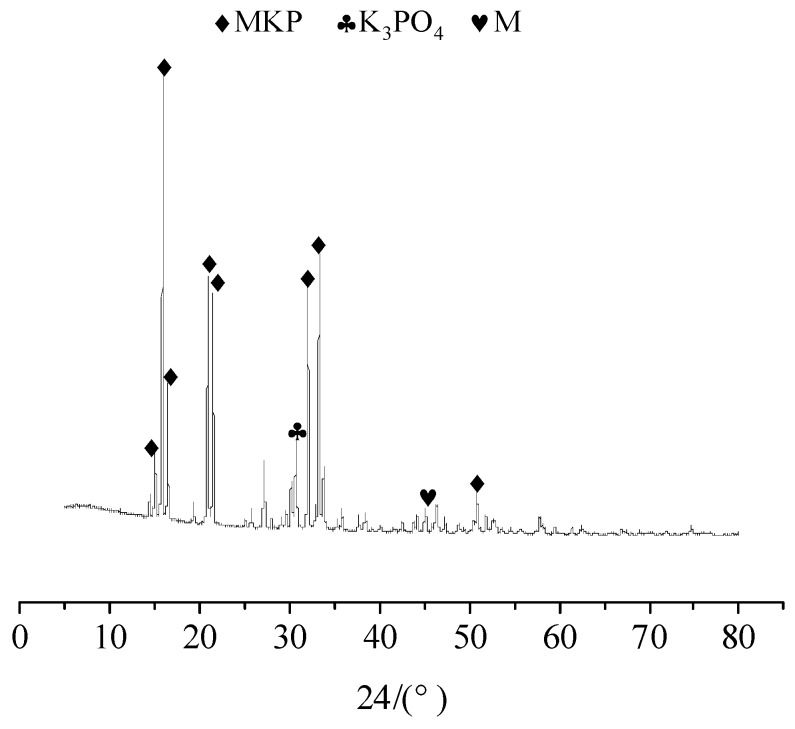
XRD pattern of the MPC mortar specimen of 7 days immersion.

Figure 8 shows the XRD spectrum of the deposits. The main extract compositions are MgKPO_4_·6H_2_O (MKP), MgO and K_3_PO_4_. The structure of MKP and MgO provides the strength of the MPC paste [60]. During the immersion, the P was dissolved and it decreased the pH value of the water solution. Therefore, the hydration reaction proceeded in the opposite direction. The MKP decomposed and the MgO dissolved in a small amount. The concentration of $K^{+}$, ${\mathrm{Mg}}^{2+}$ and ${\mathrm{PO}}_{4}^{3-}$ in the water gradually increased until the recrystallization condition of MKP was reached.

**Figure 9:**
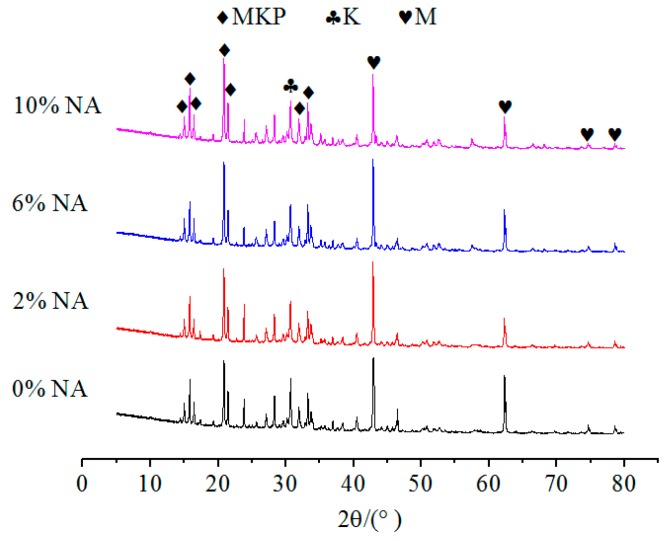
The effect of NA on the XRD patterns of MPC without immersion.

**Figure 10:**
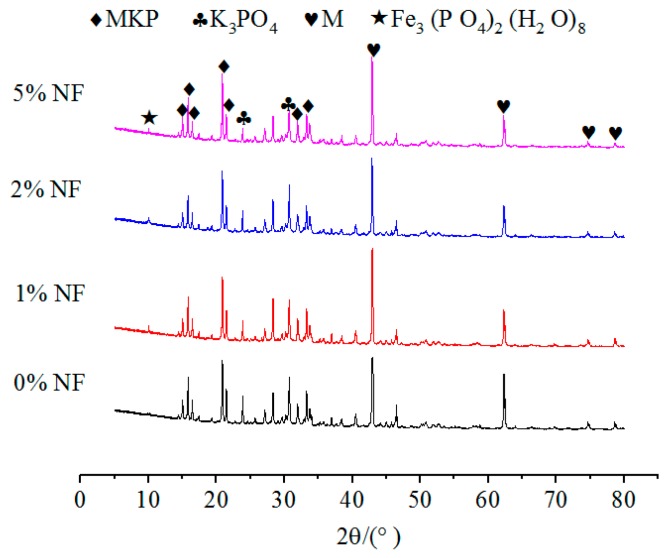
The effect of NF on the XRD patterns of MPC without immersion.

**Figure 11:**
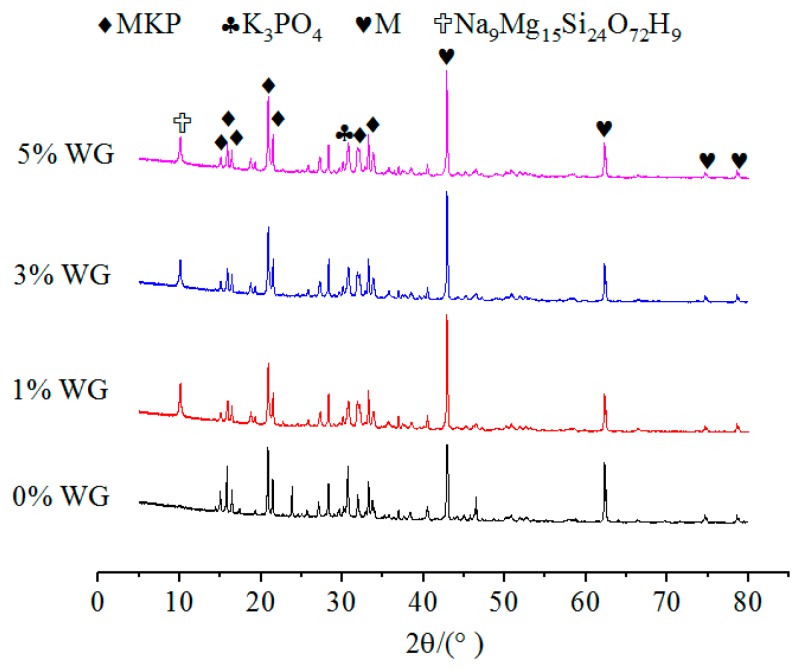
The effect of WG on the XRD patterns of MPC without immersion.

The effects of NA, NF and WG on the XRD spectrums of specimens with or without immersion were shown in Figure 9, Figure 10 and Figure 11, respectively. The relative content of MKP was significantly increased with the increasing NA from 0% to 6%, as illustrated in Figure 9, which indicates that the incorporation of NA can effectively improve the crystallinity of MKP. When the NF dosage increases from 1% to 5%, the growth peak intensity of MgO indicates that a small or excess amount of NF will decrease the crystallinity of MKP, as illustrated in Figure 10. The formation of MKP mainly depends on the following two factors:

1. pH value. When the pH value of solution is greater than 7.5, the following reaction would occur:

(2) $$\mathrm{MgO}+{\mathrm{KH}}_{2}{\mathrm{PO}}_{4}+5H_{2}O\to {\mathrm{MgKPO}}_{4}\cdot 6H_{2}O,$$

2. ${\mathrm{Mg}}^{2+}$ and ${\mathrm{PO}}_{4}^{3-}$ reach a sufficient concentration. As can be seen from Figure 8, the content of K_3_PO_4_ shows a trend of first increasing and then decreasing, which exhibits a great correlation with the MKP content. A small amount of Fe^2+^ precipitated in NF was dissolved in a solution and combined with KH_2_PO_4_ in the solution to form hydration products.

In Figure 11, the MKP content of the specimen with 1% WG subjected to 7 days of immersion is relatively higher than others, hence, the higher compressive strength of these specimens are reasonable. It is noted that the specimens with WG produced the crystals of ${\mathrm{Na}}_{9}{\mathrm{Mg}}_{15}{\mathrm{Si}}_{24}O_{72}H_{9},$ which has a positive effect on the compressive strength.

No matter the NA or NF was added or not, the types of hydration products are not affected. This is due to the fact that the main structural support inside the MPC is a stacked structure of MgO wrapped by MKP. Therefore, the relative contents of MgO and MKP, as well as the environmental conditions that promoted the formation of MKP, should be considered to judge the density of the interior structure and the compressive strength of MPC. In addition, Figure 8, Figure 9 and Figure 10 show that the curves for specimens without or with NA or NF almost have no difference. A possible situation for this reason is that the amount of NA or NF is small, and the artificial stirring error is large, so the uniformity of sampling is greatly fluctuated. In Figure 9, another new crystal was found in the XRD patterns, and the influence of NA and NF on the interior microstructure of specimens was observed by SEM. Both of Na_2_B_4_O_7_·10H_2_O and MgO were manifested in each curve in Figure 8, Figure 9 and Figure 10, which indicates that Na_2_B_4_O_7_·10H_2_O plays a positive role in retarding the ionization of MgO, contacting of phosphate and prolonging the setting time.

**Figure 12:**
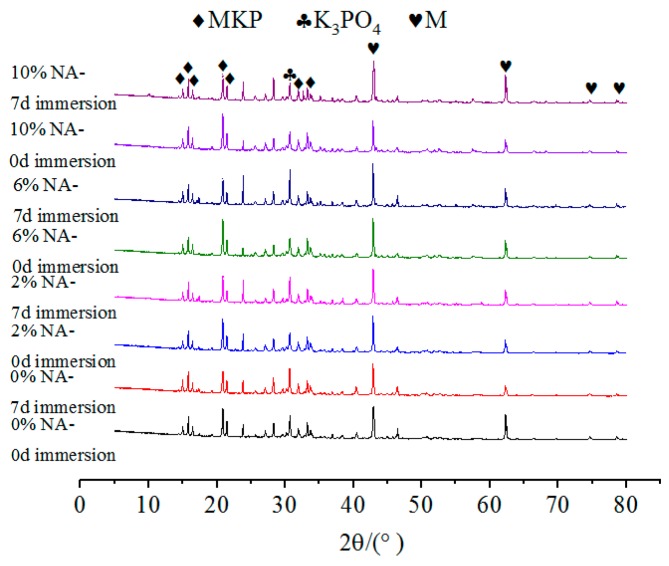
The effect of NA on the XRD patterns of MPC after 7 days of immersion.

**Figure 13:**
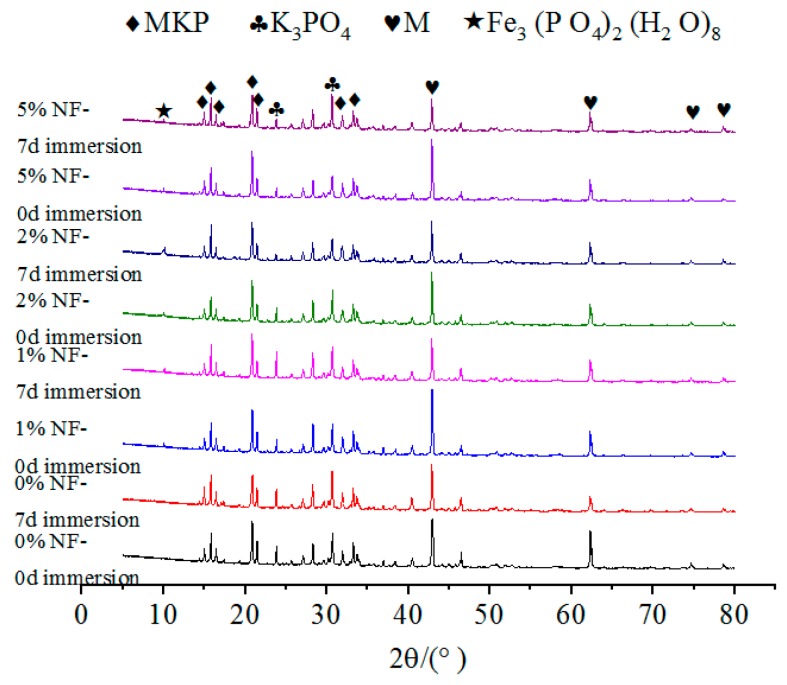
The effect of NF on the XRD patterns of MPC after 7 days of immersion.

**Figure 14:**
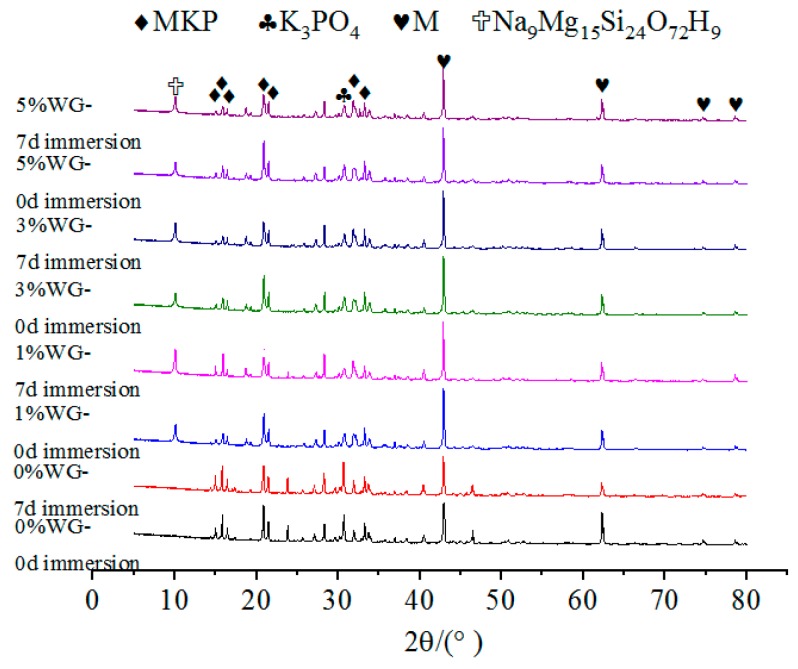
The effect of WG on the XRD patterns of MPC after 7 days of immersion.

It can be seen from Figure 12, Figure 13 and Figure 14 that for specimens with the same dosage of NA or NF, the main difference of the XRD spectrum is reflected in the relative content of MgO and MKP. After 7 days of immersion, the relative content of MKP decreased to a certain extent. This also confirms that the water stability of MPC is related to the dosages of NA or NF.

3.3 Micrograph Analysis Based on SEM Tests

**Figure 15:**
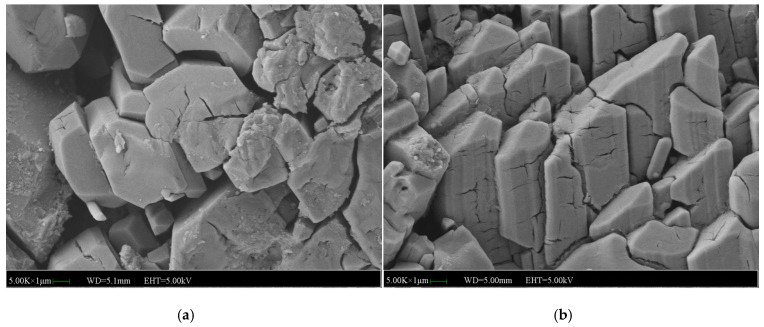
SEM images of the MPC mortar without NA, NF and WG: (**a**) SEM image of the MPC mortar without immersion, (**b**) SEM image of the MPC mortar immersed for 7 days.

Figure 15 shows the micromorphology of the specimens without NA, NF and WG. It can be seen from Figure 15a that a large number of columnar and plate-like bodies of MKP crystals were crossing each other to form a network structure for the specimens without immersion. After 7 days of immersion, the MKP crystals expanded and a few of the fine cracks among the MKP crystals began to propagate in depth and width until the network structure was destroyed, as shown in Figure 15b. It was noticed that the expansion of the cracks usually started from the large gaps among the MKP crystals, which indirectly indicates that the compactness of the matrix and the MKP crystals arrangement determines the water stability. It can be concluded that the microstructure of specimens without NA, NF and WG that were subjected to 7 days of immersion will be broke down and eventually lead to the weakness of compressive strength.

**Figure 16:**
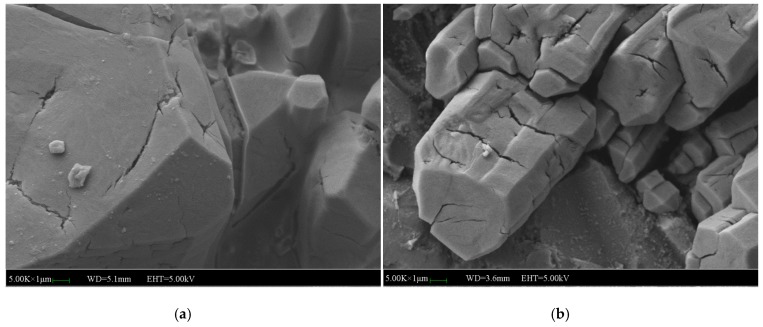
SEM images of the MPC mortar with 6% NA: (**a**) SEM image of the MPC without immersion, (**b**) SEM image of the MPC immersed for 7 days.

**Figure 17:**
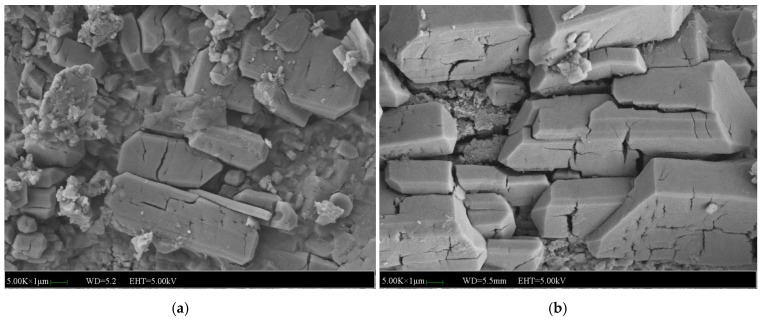
SEM images of the MPC mortar with 10% NA: (**a**) SEM image of the MPC without immersion, (**b**) SEM image of the MPC immersed for 7 days.

**Figure 18:**
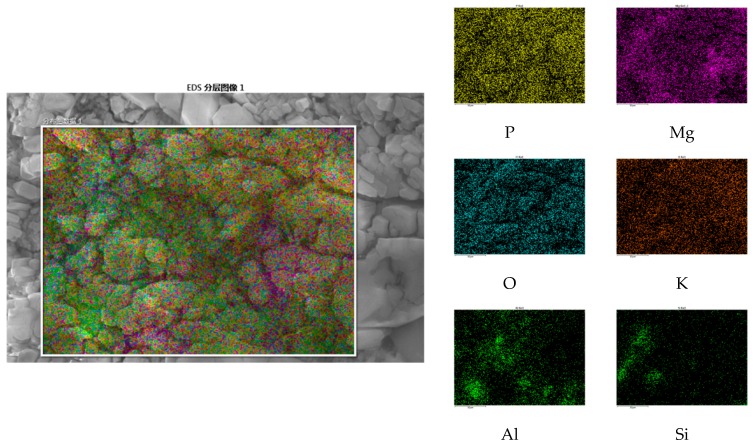
Layered image of the MPC mortar with 6% NA.

Figure 16 and Figure 17 shows the micromorphology of specimens with 6% and 10% NA, respectively. The interface between the MKP crystals is well occluded. Compared with specimens without NA, the specimens with 6% and 10% NA have less cracks of the matrix. The difference between 6% and 10% is that the distribution of cracks in the hydration products after water immersion has insignificant regularity, indicating that the effect of NA on the MKP crystal interface and the crystal itself is not much different, and has a good promotion effect. Energy Dispersive Spectroscopy (EDS) detection found that the NA distribution in the structure is relatively uniform, as shown in Figure 18. This is directly related to the uniformity of the distribution of cracks in the microstructure.

**Figure 19:**
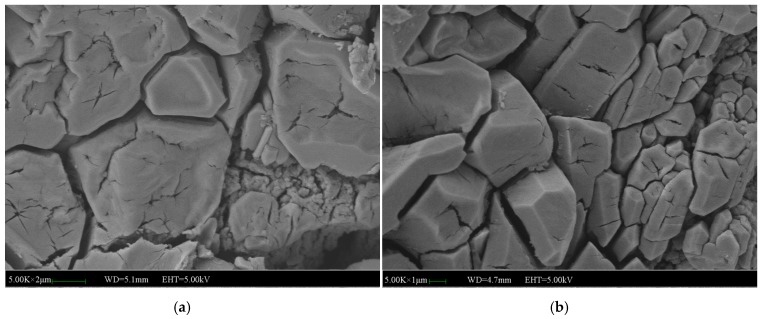
SEM images of the MPC mortar with 2% NF: (**a**) SEM image of the MPC without immersion, (**b**) SEM image of the MPC immersed for 7 days.

**Figure 20:**
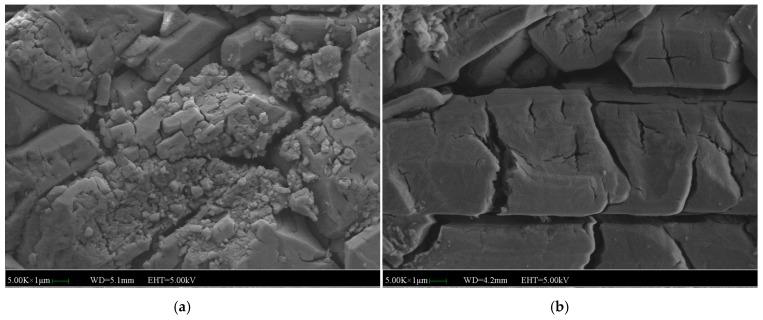
SEM images of the MPC mortar with 5% NF: (**a**) SEM image of the MPC without immersion, (**b**) SEM image of the MPC immersed for 7 days.

Figure 19 and Figure 20 shows the micromorphology of specimens with 2% and 5% NF. The microstructure of specimens with 2% NF is more compact than that of specimens without and with 5% NF. It indicates that 2% NF improved the compactness of the microstructure and enhanced the bond among the crystals. The hydration product (MKP) of the matrix has high crystallinity, and the crystal morphology is no longer a single columnar body. The occlusal interface of the crystal bodies became dense, and the micro-cracks perpendicular to the interface at the occlusal interface are less. The hydration is relatively intact, and the surrounding crystals are closely connected to each other. More NF is uniformly dispersed between the surface and the gap, which increases the compactness of the matrix. After 7 days of immersion, the surface of the columnar body formed similar microcracks to the reference group. It can be seen that the micro-cracks at the junction of the crystal body and the MgO particles and the columnar surface covered with more NF particles are less. The direction and the initial point of the crack are quite different from those of the reference group. There are many cracks extending from a certain point inside the crystal, and fewer defectsare developed from the crack between the crystals. It is indicated that the addition of NF provides a positive effect on the adhesion between crystals, but does not substantially change the internal structural properties of the crystal. Moreover, the number of micro-cracks added with NF is also greatly reduced. Therefore, in the water environment, the development of crystal cracks has been significantly inhibited, and the macroscopic water stability is enhanced.

**Figure 21:**
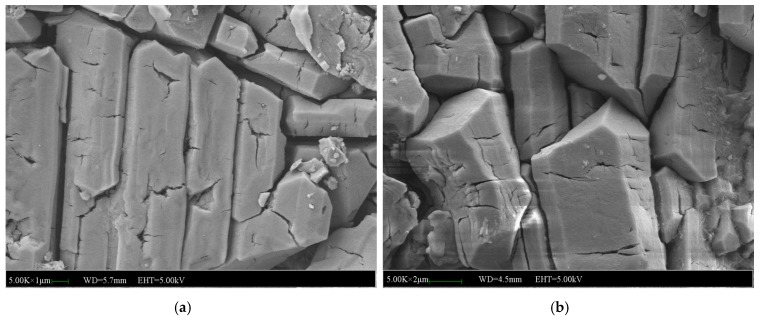
SEM images of the MPC mortar with 1% WG: (**a**) SEM image of the MPC without immersion, (**b**) SEM image of the MPC immersed for 7 days.

**Figure 22:**
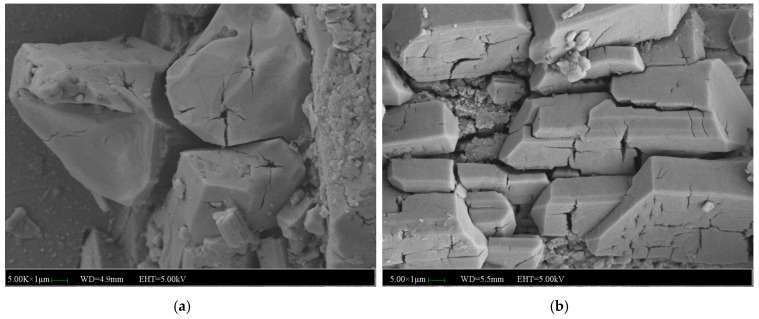
SEM images of the MPC mortar with 3% WG: (**a**) SEM image of the MPC without immersion, (**b**) SEM image of the MPC immersed for 7 days.

Figure 21 and Figure 22 show the appearances of MPC with 1% and 3% WG after 7 days of immersion or not. Compared with the specimens with 3% WG, the specimens with 1% WG looks dense enough and has mass of crystals. Besides, the MKP bonded tightly. As a result, the strength and water stability is higher.

## 4. Conclusions

In this study, the effects of NA, NF and WG on the compressive strength and water stability of MPC was experimentally investigated. The micrograph and composition of the MPC modified by NA, NF and WG were measured using SEM and XRD, respectively. Based on the experimental and analytical investigations, the following conclusions can be drawn:

1. The appropriate dosage of NA, NF and WG significantly improved the compressive strength and water stability of MPC mortar. For the water stability, the optimal dosages of NA, NF and WG are 10%, 2% and 5%, respectively. After 28 days of immersion, the strength retention rate for MPC mortar with the optimal dosages of NA, NF and WG are 86%, 101% and 96%, respectively.
2. The microstructure of the MPC mortar with the appropriate amount of NA, NF and WG is denser than that of the reference group, the micro cracks between the MKP crystals are smaller, and the crack direction and the crystal interface occlusion were different from the reference group. That resulted in the developed water stability of MPC modified by NA, NF and WG.
3. After 7 days of immersion, the hydration products of MKP become less. For amounts of MKP, the optimal dosages of NA, NF and WG are 10%, 2% and 1%. The NA, NF and WG are beneficial to increase the crystallinity of hydrated products and improve the compactness and water stability of the structure.

## Figures and Tables

**Table 1:** The properties of the materials used in this study.

Materials	Particle Size	Specific Surface Area	Appearance	Main Content	Manufacturer
Re-burned magnesia MgO (M)	-	315.7 m^2^/kg	Light yellow powder	≥97.0%	Huanai [50]
Potassium dihydrogen phosphate KH_2_PO_4_ (P)	180–600 μm	-	White crystal	≥99.0%	Weitong [51]
Retarder Na_2_B_4_O_7_·10H_2_O (B)	80–220 μm	-	White powder	≥99.5%	Banda [52]
Nano-Al_2_O_3_ (NA)	30–40 nm	200 m^2^/g	White powder	≥99.0%	Zhitai [53]
Nano-Fe_2_O_3_ (NF)	20–50 nm	80–90 m^2^/g	Red powder	≥99.8%	Zhitai [53]
Water glass Na_2_SiO_3_·5H_2_O (WG)	-	-	White powder	≥99.0%	Damao [54]

Note: The absent content is not tested and offered by the manufacturer.

**Table 2:** Mixture proportions and test variables of Magnesium Phosphate Cement (MPC) mortar/paste.

Particles Type	Particles (%)	MPC (%)	*s*/*b*	Water (%)	*m*/*p*	Immersion Time
None	0	100	0/0	16	4	0 day/7 days/28 days (Water stability, X-Ray Diffraction (XRD) analysis and Scanning Electron Microscope (SEM) observation)
			50/0			
			100/0			
			120/0			
			100/0	12	4	
			100/0	14		
			100/0	16		
			100/0	18		
			100/0	16	3	
			100/0		4	
			100/0		5	
			100/0		6	
NA	2	98	100/0	16	4	0 day/7 days/28 days (Water stability); 0 day/7 days (XRD analysis and SEM observation)
	4	96				
	6	94				
	8	92				
	10	90				
NF	1	99	100/0	16	4	
	2	98				
	3	97				
	4	96				
	5	95				
WG	1	99	100/0	16	4	
	2	98				
	3	97				
	4	96				
	5	95				

**Table 3:** Appearance of MPC mortar with NA before and after immersion.

NA Dosage					
	2%	4%	6%	8%	10%
Without immersion	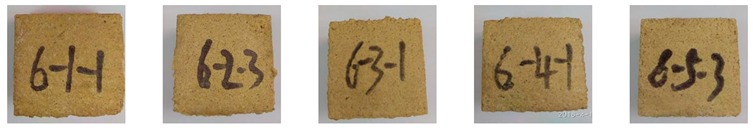				
7 days immersion	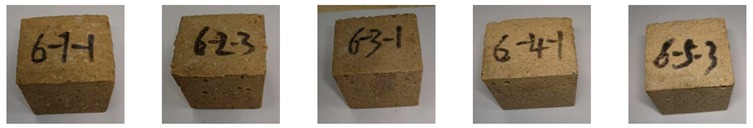				
28 days immersion	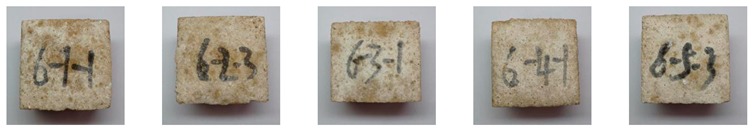				

**Table 4:** Appearance of MPC mortar with NF before and after immersion.

NF Dosage					
	1%	2%	3%	4%	5%
Without immersion	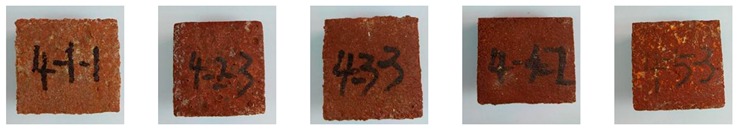				
7 days immersion	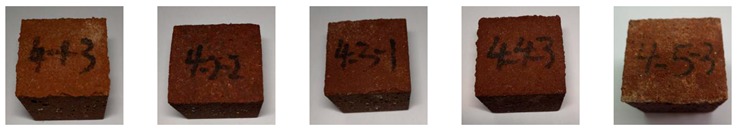				
28 days immersion	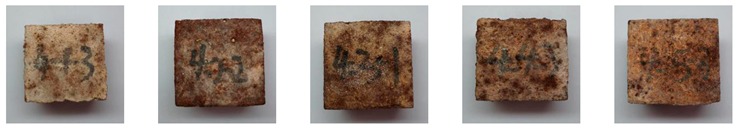				

**Table 5:** Appearance of MPC mortar with WG before and after immersion.

WG Dosage					
	1%	2%	3%	4%	5%
Without immersion	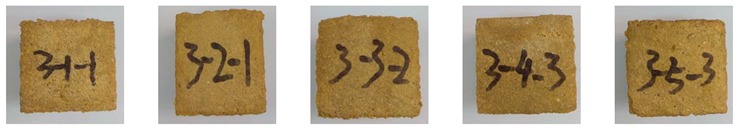				
7 days immersion	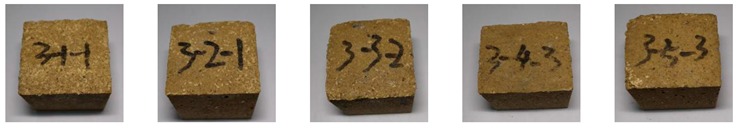				
28 days immersion